# Comparative Analysis of Gut Bacteria of Four Waterbirds Species in Taolimiao‐Alashan Nur (T‐A Nur) in Erdos Relic Gull National Nature Reserve, Inner Mongolia, China

**DOI:** 10.1002/ece3.71432

**Published:** 2025-05-13

**Authors:** Mingxin Zou, Xuanyu Li, Chunyu Li, Hongda Pei, Ruobing Kang, Li Liu, Li Gao

**Affiliations:** ^1^ College of Ecology and Environment Baotou Teacher's College Baotou China

**Keywords:** Black‐necked Grebe ( *Podiceps nigricollis* ), Erdos Relic Gull national nature reserve, Greylag Goose ( *Anser anser* ), gut bacteria, Relict Gull (*Larus relictus)*, Ruddy Shelduck ( *Tadorna ferruginea* )

## Abstract

Taolimiao‐Alashan Nur (T‐A Nur) is an important breeding site for the Relict Gulls ( *Larus relictus* ) and many other waterbirds. To understand the gut health status of rare bird species living there and to protect these bird species, this study analyzed the gut microbiota of four waterbird species, including Relict Gull ( *L. relictus* ), Black‐necked Grebe ( *Podiceps nigricollis* ), Greylag Goose ( *Anser anser* ), and Ruddy Shelduck ( *Tadorna ferruginea* ), using 16S rRNA high‐throughput sequencing. Results showed that the gut microbiota of Ruddy Shelduck had the highest α‐diversity, while Greylag Goose had the lowest. The composition of gut microbiota varied significantly among the bird species. The dominant bacterial phylum in the guts of Black‐necked Grebe, Greylag Goose, and Ruddy Shelduck was Firmicutes, while it was Pseudomonadota in Relict Gull. At the genus level, the dominant bacteria were *Halomonas* in Black‐necked Grebe, *Escherichia‐Shigella* in Relict Gull, *Ligilactobacillus* in Greylag Goose, and *Enterococcus* in Ruddy Shelduck. Correlation analysis revealed significant relationships among gut bacterial communities, suggesting that gut bacteria can regulate host metabolism and physiological state by their interactions. KEGG functional predictions indicated that gut microbiota were primarily involved in metabolism. The abundance of metabolism‐related microorganisms in Relict Gull was significantly lower than in Greylag Goose and Ruddy Shelduck, indicating that the gut microbiota of Greylag Goose and Ruddy Shelduck can provide stronger metabolic functions for the hosts. Additionally, microorganisms related to human diseases were more abundant in the gut of Relict Gull compared to Ruddy Shelduck and Black‐necked Grebe, and in Greylag Goose compared to Ruddy Shelduck. These findings suggested that the gut microbiota of birds in this area harbor some human pathogens, which warrants attention and preventive measures.

## Graphical abstract

This study analyzed the gut microbiota of four waterbird species including Relict Gull (*Larus relictus*), Black‐necked Grebe (*Podiceps nigricollis*), Greylag Goose (*Anser anser*), and Ruddy Shelduck (*Tadorna ferruginea*) using 16S rRNA high‐throughput sequencing in T‐A Nur in Erdos Relic Gull National Nature Reserve, Inner Mongolia, China to understand the survival status of rare bird species living there.

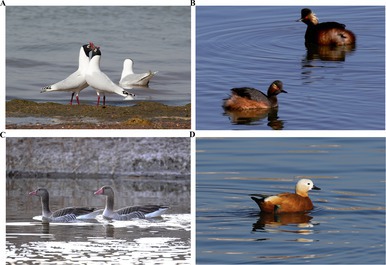

## 1 Introduction

Taolimiao‐Alashan Nur (T‐A Nur) is located in the western Ordos Plateau in China and was once a desert saline lake with an area of 10 km^2^. In the 1990s, this region supported the largest breeding population of Relict Gulls ( *Larus relictus* ) and other migratory waterbirds, earning it recognition as the 1148th Wetland of International Importance (Song et al. 2023). However, due to severe human disturbances and prolonged drought, T‐A Nur gradually dried up, eventually leading to the complete disappearance of migratory waterbirds (He et al. 2018). To restore the ecological environment of this critical wetland and facilitate the return of Relict Gulls, the local government officially launched the Yellow River Water Diversion Project in May 2018, supplying water to T‐A Nur (He et al. 2019). By April 2019, breeding populations of Relict Gulls had returned to the area, revitalizing T‐A Nur with its former vibrancy (He et al. 2019). In the short term, we have observed the positive impact of the water diversion project on wetland restoration and biodiversity enhancement, particularly for the bird species inhabiting the area. For them, the water source provided by T‐A Nur is essential for survival. However, the water supplied through the diversion project originates from mine drainage. Studies have shown that mine water contains significant heavy metal pollutants (McDevitt et al. 2020), which can pose serious threats to living organisms. Therefore, evaluating the health status of waterbirds inhabiting this area is of great importance.

Gut microbiota are a critical component of animal health. Microorganisms provide hosts with numerous essential functions, including aiding in digestion, synthesizing vitamins, defending against pathogens, enhancing immune capacity, and supporting organ development (Qin et al. 2010; Diaz et al. 2011; Al‐Asmakh et al. 2014). They can even influence host behavior (Cryan and Dinan 2012). With increasing awareness of environmental conservation, there is growing attention to biodiversity protection. Meanwhile, birds occupy an important ecological niche in ecosystems and are well‐known hosts of emerging human infectious diseases (Mackenzie and Jeggo 2013). Bird migration facilitates the spread of pathogens across multiple geographic ranges (Mackenzie and Jeggo 2013). Therefore, research on bird gut microbiota plays an important role in the conservation and management of wild bird populations, as well as providing insights for preventing the spread of pathogenic microorganisms. To date, the gut microbiota of many rare bird species has drawn significant attention. For example, studies have reported on the gut microbiota of the Jankowski's bunting ( *Emberiza jankowskii* ) (Fernando et al. 2010) and the whooper swan ( *Cygnus cygnus* ) (Guan et al. 2017). These studies provide insights into the conservation and management of rare species.

**FIGURE 1:**
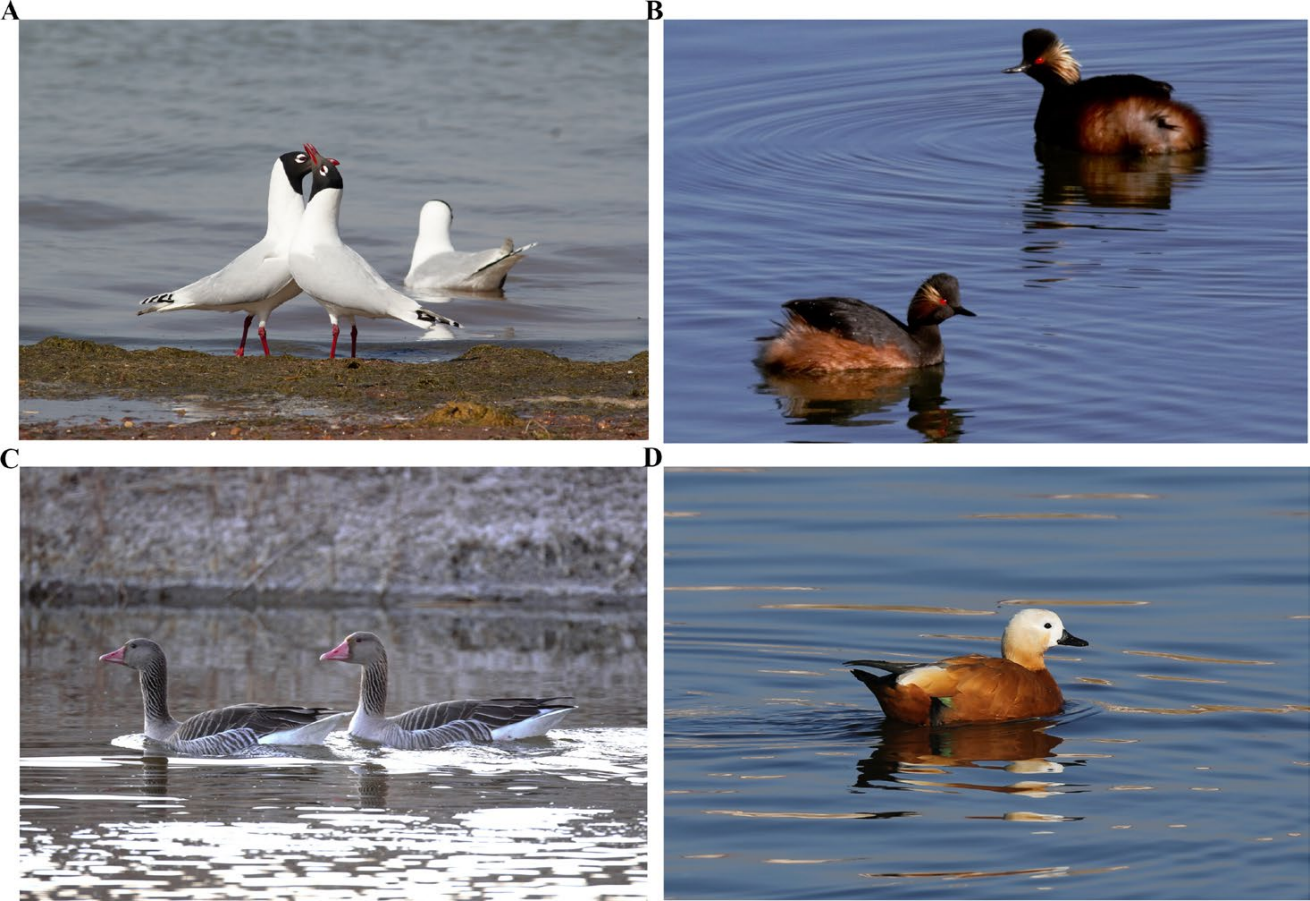
Organism photograph. (A) Relict Gull ( *Larus relictus* ); (B) Black‐necked Grebe ( *Podiceps nigricollis* ); (C) Greylag Goose ( *Anser anser* ); (D) Ruddy Shelduck ( *Tadorna ferruginea* ).

Based on the above background, the present study focuses on four bird species inhabiting T‐A Nur, including the Relict Gull ( *L. relictus* ), Black‐necked Grebe ( *Podiceps nigricollis* ), Greylag Goose ( *Anser anser* ), and Ruddy Shelduck ( *Tadorna ferruginea* ) (Figure 1). Among these species, the Relict Gull is listed as a national first‐class protected species, while the Black‐necked Grebe and Greylag Goose are listed as national second‐class protected species. By analyzing the gut microbiota of these rare species using 16S rRNA high‐throughput sequencing, this study will contribute valuable insights into their gut health status, indirectly reflecting their physiological condition while inhabiting the region. Moreover, by monitoring the gut microbiota, potential pathogenic bacteria carried by the waterfowl can be identified, providing a reference for management authorities to establish effective protective measures and thereby prevent the spread of pathogenic bacteria.

## 2 Materials and Methods

### 2.1 Study Area and Sample Collection

T‐A Nur (E 109°14′‐109°23′; N 39°45′‐39°52′) is located in Dongsheng District, Ordos City, Inner Mongolia, China. From May to December 2021, fresh fecal samples from four bird species were collected in the Ordos Relict Gulls National Nature Reserve. Before sampling, the habitats of different bird species were monitored with binoculars. Once a target was identified, the fresh fecal samples were collected around the birds' habitat islands. Surface debris and the parts in contact with the ground were removed, and the samples were stored in paper bags. To avoid cross‐contamination, gloves were changed after collecting each sample. A total of 21 samples were collected, including 6 from Relict Gull, 5 from Black‐necked Grebe, 5 from Greylag Goose, and 5 from Ruddy Shelduck. The samples were transported on dry ice to the laboratory for about 2 h and then stored at −80°C for further analysis (Liu et al. 2022).

### 2.2 DNA Extraction, PCR Amplification, and High‐Throughput Sequencing

Total DNA was extracted from 1 g fecal samples of each bird using a fecal DNA extraction kit (Stool DNA Kit (Tiangen Biotech (Beijing) Co. Ltd.), DP328). The hypervariable V3‐V4 regions of the bacterial 16S rRNA gene were amplified using primers 341F (5’‐CCTACGGGNGGCWGCAG‐3′) and 805R (5’‐GACTACHVGGGTATCTAATCC‐3′). The polymerase chain reaction (PCR) procedure was as follows: predenaturation at 94°C for 5 min; denaturation at 94°C for 30 s; annealing at 55°C for 30 s; extension at 72°C for 45 s, 35 cycles; extension at 72°C for 10 min; and storage at 4°C. The purified DNA was sequenced using the Illumina NovaSeq platform by Beijing Biomarker Technologies Co. Ltd. (Beijing, China).

### 2.3 Sequence Analysis

The bioinformatics analysis of this study was performed with the aid of the BMKCloud (http://www.biocloud.net/). After quality control and filtering of sequencing data, operational taxonomic units (OTUs) were clustered at a 97% sequence similarity threshold, and the community composition of each sample was analyzed at various taxonomic levels by the Silva 138 database (https://www.arb‐silva.de/documentation/release‐138/) (Quast et al. 2013) with RDP Classifier software, with a confidence threshold of 0.8. Alpha diversity was assessed using the Chao1, ACE, Simpson, and Shannon indices and calculated using the Qiime2 software (Version 2020.6.0, https://qiime2.org) (Caporaso et al. 2010), significance was tested using one‐way analysis of variance. Beta diversity was analyzed using Bray–Curtis NMDS and Binary Jaccard NMDS using Qiime2 software (Version 2020.6.0, https://qiime2.org) (Caporaso et al. 2010), and significance was tested using ANISOM. Differential bacteria between groups were identified using LEfSe (Segata et al. 2011) (Linear Discriminant Analysis (LDA) Effect Size) (Version 1.1.1, https://github.com/SegataLab/lefse/tree/master/lefse) with thresholds of LDA > 4.0 and *p* < 0.05. Spearman's correlation was performed to evaluate associations among gut bacteria using the OmicShare tool, a free online platform for data analysis (https://www.omicshare.com/tools). The KEGG database was used to predict the functional profiles of gut bacteria using the Picrust2 software (Version 2.3.0, https://huttenhower.sph.harvard.edu/picrust) (Douglas et al. 2020).

## 3 Results

### 3.1 Sequence Statistics and Alpha Diversity Analysis of Gut Microbiota in Four Bird Species

The gut microbiota of Relict Gull (YO), Black‐necked Grebe (PT), Greylag Geese (HY), and Ruddy Shelduck (CMY) were analyzed through 16S rRNA V3‐V4 region amplicon sequencing. A total of 1,684,674 sequences were obtained from 21 samples, including 6 Relict Gull samples, 5 Black‐necked Grebe samples, 5 Greylag Goose samples, and 5 Ruddy Shelduck samples. After filtering, 1,649,659 high‐quality sequences were retained, accounting for 97.92% of the total sequences. The high accuracy of sequencing meets analytical standards. From these 21 samples, a total of 6894 OTUs were identified. The number of OTUs obtained from each sample is shown in Table 1.

**TABLE 1:** Basic information on high‐throughput sequencing of gut bacteria 16S rRNA genes in the four bird species.

Sample	Raw reads	Clean reads	Effective rate (%)	Number of OTUs	Number of taxa of different taxonomic categories				
					Phylum	Class	Order	Family	Genus
PT1	79,918	79,726	0.9976	344	15	23	58	126	203
PT2	79,916	79,750	0.9979	437	20	36	76	144	215
PT3	79,920	79,748	0.9978	298	17	26	56	112	173
PT4	80,357	80,192	0.9979	260	17	25	59	113	164
PT5	80,375	80,156	0.9973	560	22	40	89	178	299
YO1	80,181	80,014	0.9979	112	11	13	26	46	75
YO2	79,904	79,724	0.9977	575	21	34	84	145	263
YO3	80,038	79,858	0.9976	573	17	34	82	152	282
YO4	80,228	80,036	0.9976	661	22	42	104	178	324
YO5	79,867	79,690	0.9978	200	13	20	51	83	131
YO6	79,792	79,621	0.9979	374	19	31	70	121	198
HY1	80,103	73,915	0.9227	455	22	36	97	182	302
HY2	79,808	73,324	0.9188	306	17	27	74	133	215
HY3	80,231	74,419	0.9276	197	17	25	56	93	142
HY4	79,778	73,906	0.9264	405	20	37	91	152	246
HY5	83,912	77,090	0.9187	545	24	43	110	202	345
CMY1	80,239	79,860	0.9953	413	19	35	73	118	188
CMY2	80,286	79,894	0.9951	625	24	39	95	168	279
CMY3	80,081	79,723	0.9955	857	24	51	125	222	384
CMY4	79,988	79,628	0.9955	683	20	31	71	118	211
CMY5	79,752	79,385	0.9954	590	21	41	105	178	287

Abbreviations: CMY, Ruddy Shelduck; HY, Greylag Goose; PT, Black‐necked Grebe; YO, Relict Gull.

**FIGURE 2:**
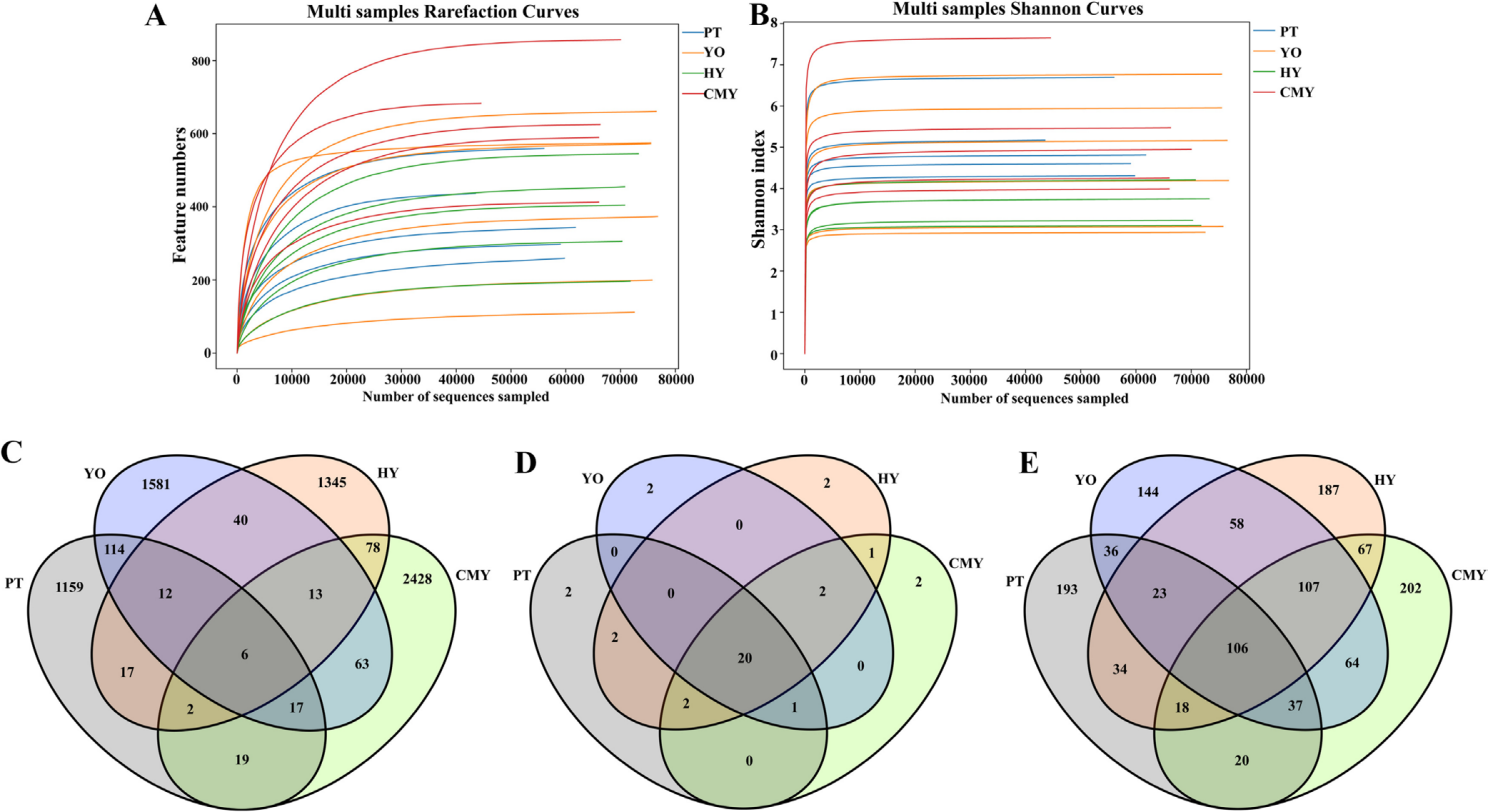
Sequencing data statistics. (A) Rarefaction curve; (B) Shannon curve; (C) OTU distribution for each group; (D) Phylum‐level bacterial distribution for each group; (E) Genus‐level bacterial distribution for each group. CMY, Ruddy Shelduck; HY, Greylag Goose; PT, Black‐necked Grebe; YO, Relict Gull.

For the 21 fecal samples collected from Relict Gull, Black‐necked Grebe, Greylag Geese, and Ruddy Shelduck, rarefaction curves and Shannon index curves for 16S rRNA sequencing reached a plateau (Figure 2A,B). This indicates that microbial diversity did not increase with deeper sequencing, confirming that the sequencing depth was sufficient to reflect the majority of microbial diversity in the samples.

A Venn diagram was used to compare the diversity of bacterial communities among the bird species. At the OTU level, six OTUs were shared among all four bird species, while each species had two unique OTUs (Figure 2C). At the phylum level, 20 bacterial phyla were shared among the four bird species, and each species had two unique bacterial phyla (Figure 2D). At the genus level, 106 bacterial genera were shared across all four bird species. In terms of unique genera, Relict Gull had 144, Greylag Goose had 187, Black‐necked Grebe had 193, and Ruddy Shelduck had 202 (Figure 2E).

**FIGURE 3:**
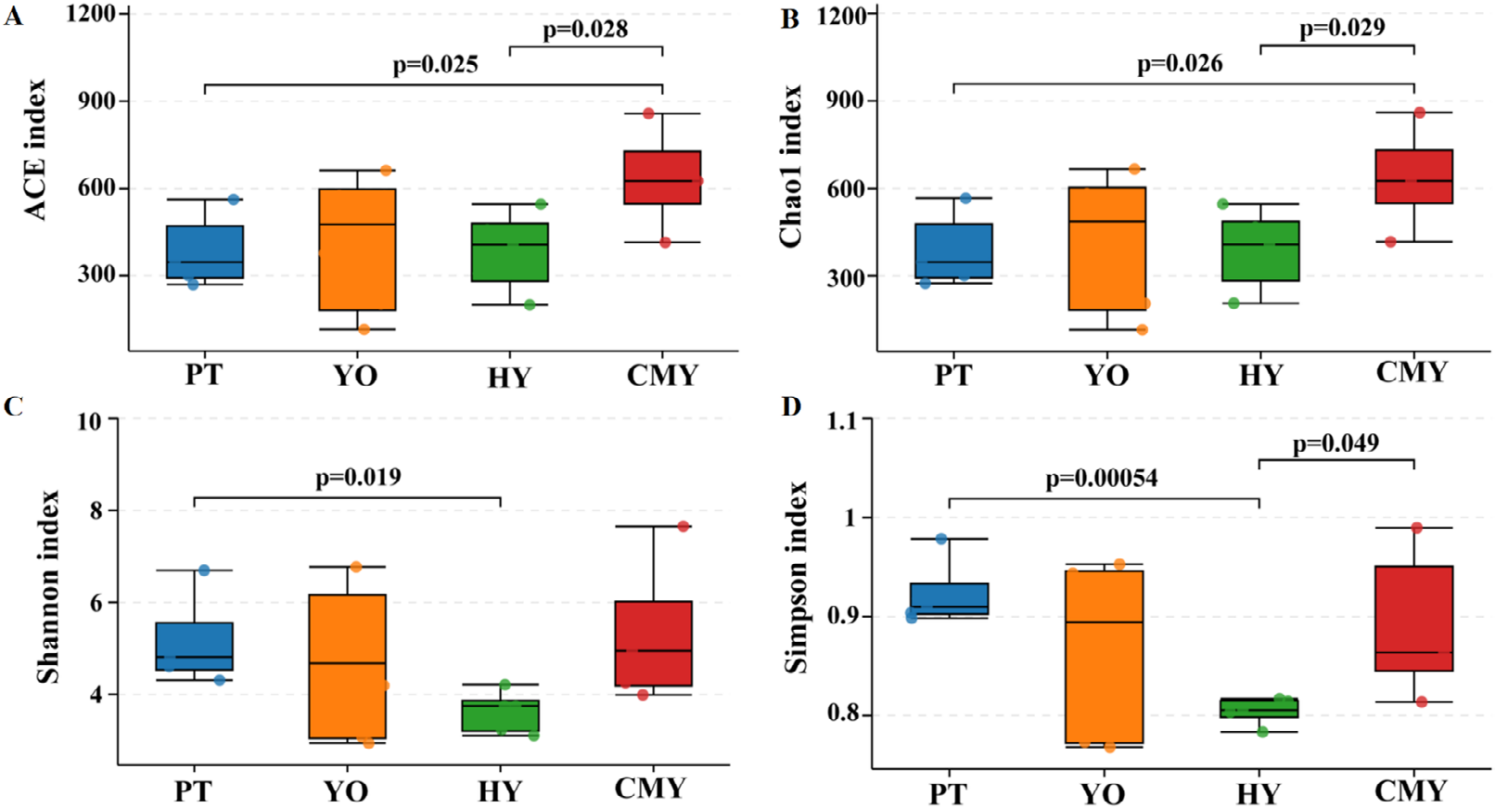
α‐Diversity indices (A) ACE index; (B) Chao1 index; (C) Shannon index; (D) Simpson index. CMY, Ruddy Shelduck; HY, Greylag Goose; PT, Black‐necked Grebe; YO, Relict Gull.

At the same sequencing depth, alpha diversity indices, including ACE, Chao1, Shannon, and Simpson, were analyzed to compare bacterial diversity across the bird species. Significant differences in gut bacterial diversity were observed among the species. For the ACE and Chao1 indices, Ruddy Shelduck was significantly higher than Black‐necked Grebe and Greylag Goose (*p* < 0.05) (Figure 3A,B). Black‐necked Grebe had significantly higher Shannon and Simpson indices than Greylag Goose (*p* < 0.05), and Ruddy Shelduck had a significantly higher Simpson index than Greylag Goose (*p* < 0.05) (Figure 3C,D). In summary, Ruddy Shelduck exhibited the highest gut microbial alpha diversity, while Greylag Goose showed the lowest.

### 3.2 Beta Diversity Analysis of Gut Microbiota in Four Bird Species

**FIGURE 4:**
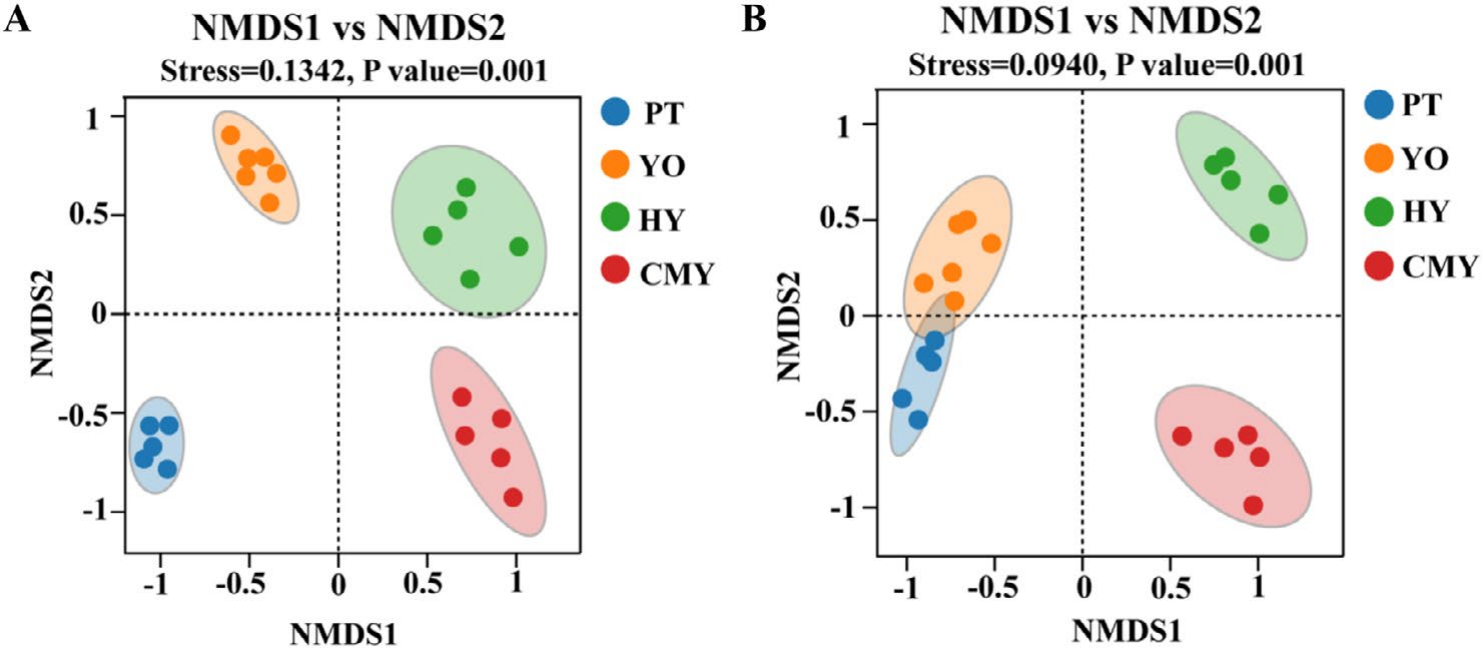
α‐Diversity analysis. (A) Bray–Curtis NMDS; (B) Binary Jaccard NMDS. CMY, Ruddy Shelduck; HY, Greylag Goose; PT, Black‐necked Grebe; YO, Relict Gull.

Beta diversity of bacterial communities across different samples was analyzed using Bray–Curtis NMDS and Binary Jaccard NMDS. The results, shown in Figure 4A,B, indicated a significant separation trend between different groups, while samples within the same group exhibited significant clustering trends. ANOSIM tests further confirmed that the beta diversity differences between groups were highly significant (*p* < 0.01).

### 3.3 Gut Microbial Community Composition Analysis in Four Bird Species

**FIGURE 5:**
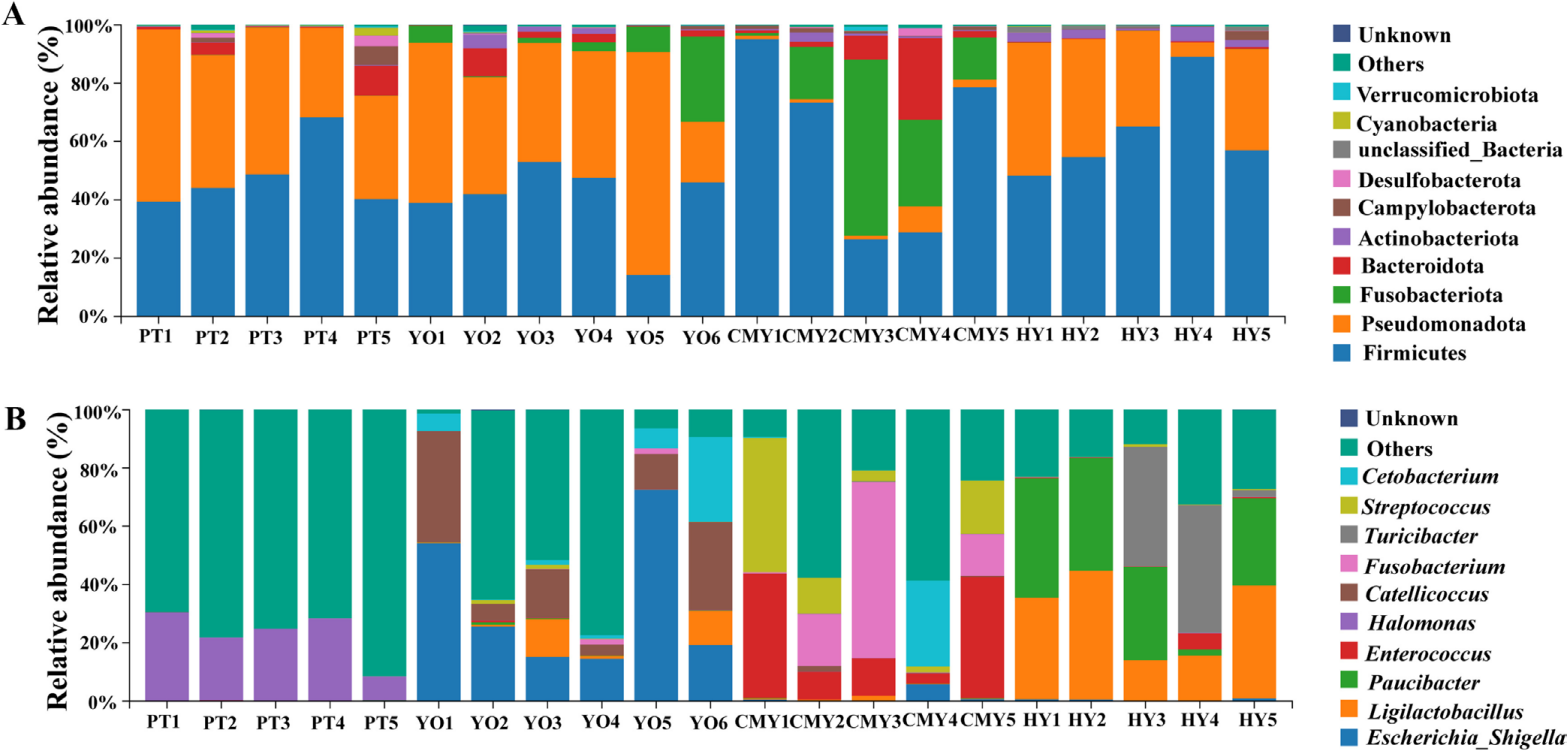
Bacterial composition of each sample (A) Phylum‐level bacterial composition; (B) Genus‐level bacterial composition. CMY, Ruddy Shelduck; HY, Greylag Goose; PT, Black‐necked Grebe; YO, Relict Gull.

Analysis of bacterial composition at the phylum level revealed that the most abundant phyla were Firmicutes (55.3%), Pseudomonadota (30.9%), Fusobacteriota (5.5%), Bacteroidota (4.1%), Actinobacteriota (1.4%), and Campylobacterota (1.0%). In the gut microbiota of Black‐necked Grebe, Greylag Goose, and Ruddy Shelduck, Firmicutes was the most dominant phylum, accounting for 44.2%, 62.9%, and 60.5%, respectively. In contrast, in Relict Gull, Pseudomonadota was the most dominant phylum, accounting for 46.06% (Figure 5A).

At the genus level, the top 10 most abundant bacterial genera were *Ligilactobacillus* (8.6%), *Escherichia_Shigella* (8.5%), *Enterococcus* (8.4%), *Paucibacter* (7.3%), *Halomonas* (5.7%), *Catellicoccus* (4.5%), *Turicibacter* (4.4%), *Fusobacterium* (3.5%), *Halolactibacillus* (3.3%), and *Streptococcus* (3.0%). In Black‐necked Grebe, the most abundant genus was *Halomonas*, accounting for 22.5%. In Relict Gull, the most abundant genus was *Escherichia_Shigella*, accounting for 33.6%. In Greylag Goose, the most abundant genus was *Ligilactobacillus*, accounting for 29.4%. In Ruddy Shelduck, the most abundant genus was *Enterococcus*, accounting for 22.0% (Figure 5B).

### 3.4 Differential Analysis of Gut Microbial Community Composition in Four Bird Species

**FIGURE 6:**
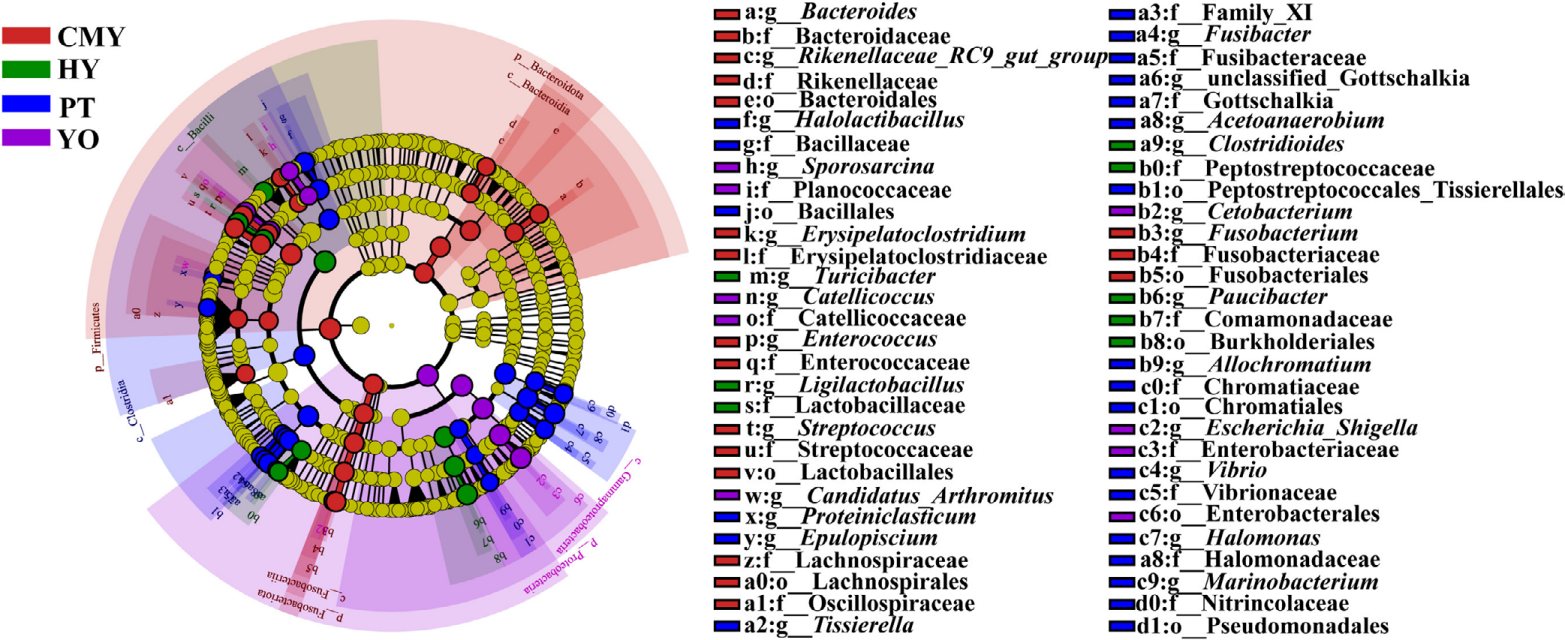
LefSe analysis of bacterial composition differences among groups. From the outer to the inner circle, classifications are shown at the genus, family, order, class, and phylum levels (LDA > 4.0, *p* < 0.05). CMY, Ruddy Shelduck; HY, Greylag Goose; PT, Black‐necked Grebe; YO, Relict Gull.

LefSe analysis was used to identify biomarker bacteria in the gut microbiota of different bird species (Figure 6). At the phylum level, the biomarker phylum in Relict Gull was Pseudomonadota, and the biomarker phyla in Ruddy Shelduck were Firmicutes, Fusobacteriota, and Bacteroidota. No biomarker phyla were identified in the gut microbiota of Black‐necked Grebe and Greylag Goose.

At the genus level, the biomarker genera in Relict Gull were *Escherichia_Shigella*, *Catellicoccus*, *Cetobacterium*, *Candidatus_Arthromitus*, and *Sporosarcina*. In Black‐necked Grebe, the biomarker genera were *Halomonas*, *Halolactibacillus*, *Fusibacter*, unclassified_Gottschalkia, *Marinobacterium*, *Vibrio*, *Epulopiscium*, *Proteiniclasticum*, *Tissierella*, *Allochromatium*, and *Acetoanaerobium*. In Greylag Goose, the biomarker genera were *Ligilactobacillus*, *Paucibacter*, *Turicibacter*, and *Clostridioides*. In Ruddy Shelduck, the biomarker genera were *Enterococcus*, *Fusobacterium*, *Streptococcus*, *Bacteroides*, *Erysipelatoclostridium*, and Rikenellaceae_RC9_gut_group (LDA > 4.0, *p* < 0.05).

### 3.5 Microbial Correlation Analysis

**FIGURE 7:**
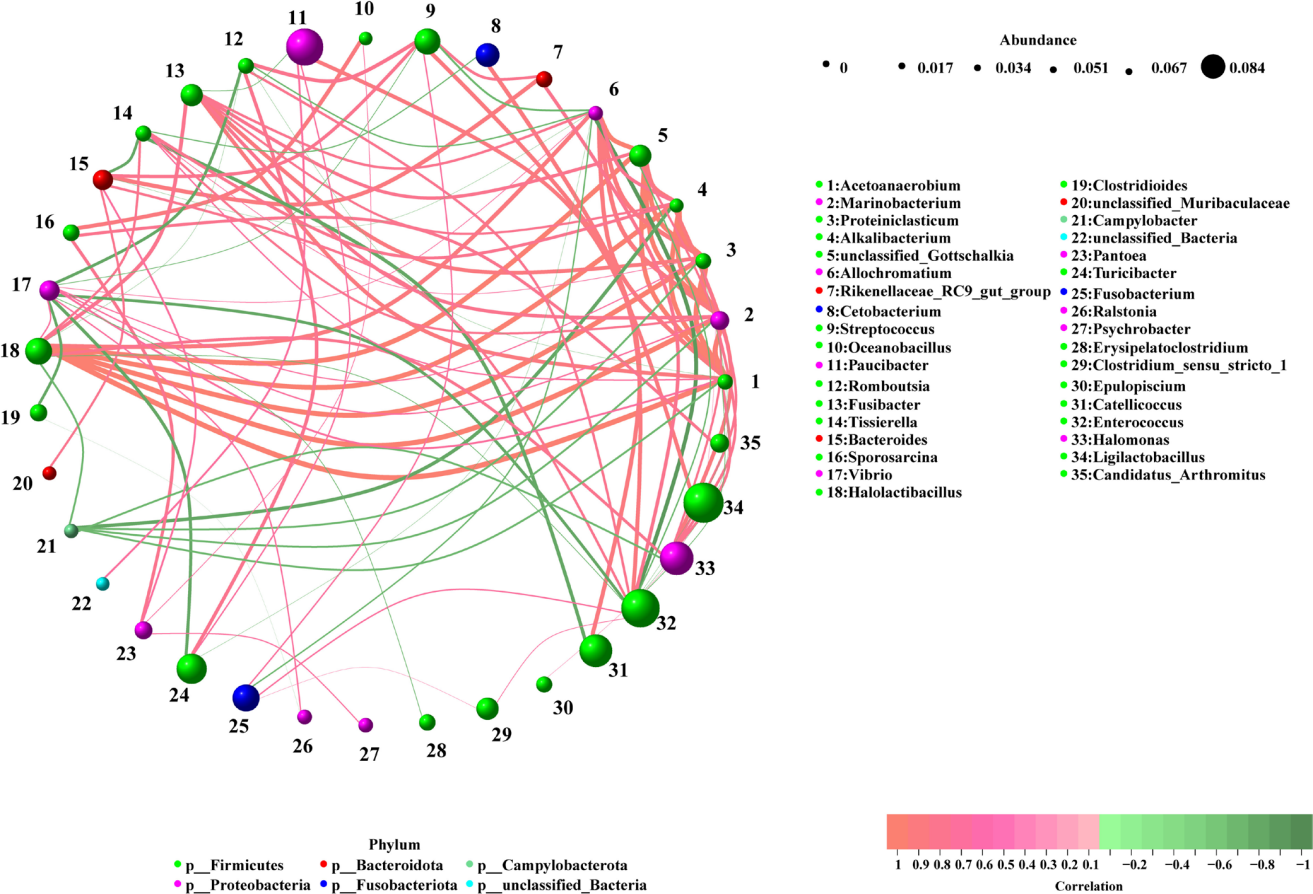
Spearman correlation analysis among gut bacteria (*p* < 0.05). The color of the nodes represents bacterial taxonomy, and the relative abundance is indicated by the weighted node size. Red lines indicate positive correlations, while green lines indicate negative correlations.

The associations between bacteria were analyzed using Spearman correlation. The results showed significant correlations among bacteria (Figure 7). The top 10 strongest correlations between bacterial pairs were as follows: *Acetoanaerobium* was highly positively correlated with *Halolactibacillus* (0.9907), *Proteiniclasticum* (0.9884), and *Marinobacterium* (0.9837) (*p* < 0.001). *Marinobacterium* was highly positively correlated with *Halolactibacillus* (0.9791, *p* < 0.001). *Proteiniclasticum* was highly positively correlated with *Marinobacterium* (0.9791) and *Halolactibacillus* (0.9697) (*p* < 0.001). *Alkalibacterium* (0.9281) and unclassified_Gottschalkia (0.9171) were highly positively correlated with *Halolactibacillus* (*p* < 0.001). *Marinobacterium* was highly positively correlated with unclassified_Gottschalkia (0.9149, *p* < 0.001). *Alkalibacterium* was highly positively correlated with *Acetoanaerobium* (0.9106, *p* < 0.001).

### 3.6 Functional Prediction Using PICRUSt2

**FIGURE 8:**
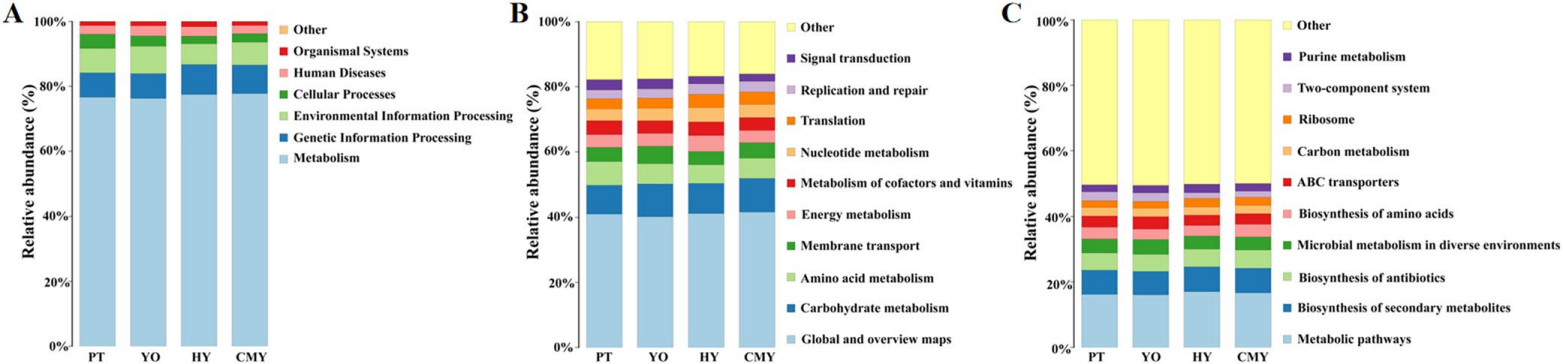
Functional prediction of gut bacteria. (A) Level 1 functional prediction; (B) Level 2 functional prediction; (C) Level 3 functional prediction. CMY, Ruddy Shelduck; HY, Greylag Goose; PT, Black‐necked Grebe; YO, Relict Gull.

The gut microbial functions were predicted using PICRUSt2 software. The results are shown in Figure 8. The predicted microbial functions were Metabolism (77.0%), Genetic Information Processing (8.3%), Environmental Information Processing (7.4%), Cellular Processes (3.1%), Human Diseases (2.8%), **and** Organismal Systems (1.4%) (Figure 8A). At Level 2, the top 10 functions were Global and overview maps (41.0%), Carbohydrate metabolism (9.5%), Amino acid metabolism (6.4%), Membrane transport (4.6%), Energy metabolism (4.1%), Metabolism of cofactors and vitamins (4.1%), Nucleotide metabolism (4.0%), Translation(3.5%), Replication and repair (3.0%), **and** Signal transduction (2.7%) (Figure 8B). At level 3, the top 10 functions were Metabolic pathways (16.5%), Biosynthesis of secondary metabolites (7.4%), Biosynthesis of antibiotics(5.4%), Microbial metabolism in diverse environments (4.2%), Biosynthesis of amino acids(3.5%), ABC transporters (3.4%), Carbon metabolism (2.6%), Ribosome(2.3%), Two‐component system(2.2%) and Purine metabolism (2.2%) (Figure 8C).

Further comparisons of bacterial functional differences between samples revealed that in the Black‐necked Grebe gut, the abundance of microbes associated with Genetic Information Processing and Cellular Processes was higher than in the Ruddy Shelduck. In the Black‐necked Grebe gut, the abundance of microbes associated with Genetic Information Processing and Organismal Systems was lower than in Greylag Goose, while those associated with Environmental Information Processing and Cellular Processes were higher than in Greylag Goose. In the Black‐necked Grebe gut, the abundance of microbes associated with Environmental Information Processing and Human Diseases was lower than in Relict Gull, while those associated with Cellular Processes were higher than in Relict Gull. In the Relict Gull gut, the abundance of microbes associated with Metabolism, Genetic Information Processing, and Organismal Systems was lower than in Greylag Goose, while those associated with Environmental Information Processing and Cellular Processes were higher than in Greylag Goose. In the Relict Gull gut, the abundance of microbes associated with Metabolism and Genetic Information Processing was lower than in Ruddy Shelduck, while those associated with Environmental Information Processing, Cellular Processes, and Human Diseases were higher than in Ruddy Shelduck. In the Greylag Goose gut, the abundance of microbes associated with Human Diseases and Organismal Systems was higher than in Ruddy Shelduck (Table 2).

**TABLE 2:** Bacterial functions that differ significantly between samples.

Function	Relative gene content (%)		
	PT	CMY	*p*
Genetic Information Processing	7.52 ± 0.46	0.26 ± 0.00	0.002526792954
Cellular Processes	4.37 ± 0.23	2.64 ± 0.18	4.45E‐06
	**PT**	**HY**	***p***
Genetic Information Processing	7.52 ± 0.46	9.24 ± 0.62	0.00246681
Environmental Information Processing	7.55 ± 0.44	6.38 ± 0.19	0.003533652
Cellular Processes	4.37 ± 0.23	2.38 ± 0.17	1.82E‐06
Organismal Systems	1.29 ± 0.1	1.64 ± 0.11	0.001349221
	**PT**	**YO**	***p***
Environmental Information Processing	7.55 ± 0.44	8.45 ± 0.6	0.029095298
Cellular Processes	4.37 ± 0.23	3.17 ± 0.39	0.000378412
Human Diseases	2.66 ± 0.15	3.15 ± 0.2	0.002101127
	**YO**	**HY**	***p***
Metabolism	76.26 ± 0.59	77.47 ± 0.57	0.012842245
Genetic Information Processing	7.64 ± 0.6	9.24 ± 0.62	0.003870999
Environmental Information Processing	8.45 ± 0.6	6.38 ± 0.19	0.000288232
Cellular Processes	3.17 ± 0.39	2.38 ± 0.17	0.004519842
Organismal Systems	1.34 ± 0.07	1.64 ± 0.11	0.002137961
	**YO**	**CMY**	***p***
Metabolism	76.26 ± 0.59	77.76 ± 0.87	0.022485823
Genetic Information Processing	7.64 ± 0.6	8.8 ± 0.26	0.005651864
Environmental Information Processing	8.45 ± 0.6	7.03 ± 0.71	0.013378742
Cellular Processes	3.17 ± 0.39	2.64 ± 0.18	0.03019633
Human Diseases	3.15 ± 0.2	2.51 ± 0.09	0.000273824
	**HY**	**CMY**	***p***
Human Diseases	2.89 ± 0.18	2.51 ± 0.09	0.009614067
Organismal Systems	1.64 ± 0.11	1.25 ± 0.04	0.001037898

Abbreviations: CMY, Ruddy Shelduck; HY, Greylag Goose; PT, Black‐necked Grebe; YO, Relict Gull.

## 4 Discussion

The Ordos Relict Gulls National Nature Reserve, also known as T‐A Nur, is one of the important breeding grounds for the Relict Gull, a nationally protected Class I species. According to bird dynamic monitoring reports, the area hosts 51 species of wetland birds, belonging to 9 orders and 13 families. Among them, two species are listed as Class I nationally protected wild animals: the Relict Gull and the Great Bustard ( *Otis tarda* ). Additionally, 13 species are listed as Class II nationally protected wild animals, including the Black‐necked Grebe and the Greylag Goose, which also breed in this area (Liu et al. 2024). The Ruddy Shelduck is the dominant species during the overwintering period in this region. Studies suggest that host species, geographic factors, environmental factors, and diet all influence the composition of gut microbiota (Valdes et al. 2018; Wu et al. 2018; Sharpton 2018; Murray et al. 2020). This study selected four bird species inhabiting the reserve to analyze their gut microbial diversity, aiming to assess the gut health status of waterbirds living in this region, offering a basis for their conservation.

The results revealed significant differences in gut microbiota diversity among the four bird species. The Ruddy Shelduck exhibited the highest gut microbiota α‐diversity, while the Greylag Goose had the lowest. This difference may be attributed to variations in diet; studies have shown that greater dietary diversity is associated with higher α‐diversity in gut microbiota (Huang et al. 2020). During the breeding season, Relict Gulls mainly feed on dragonflies and chironomid midges (Jandhyala et al. 2015), Black‐necked Grebes primarily consume invertebrates (Liu et al. 2024), Greylag Geese predominantly feed on plant leaves, roots, stems, sprouts, fruits, and seeds (Svendsen et al. 2023), and Ruddy Shelducks have a more varied diet that includes grains, insects, crustaceans, frogs, shrimp, and aquatic plants (Chang et al. 1997). Since the Greylag Goose has a relatively simple plant‐based diet, its gut microbiota α‐diversity is the lowest. In contrast, the Ruddy Shelduck, with its more diverse diet, exhibits the highest α‐diversity in gut microbiota. Studies have shown that a gut ecosystem with high‐abundance microbial communities helps hosts resist external influences and plays a critical role in preventing pathogen colonization, maintaining gut homeostasis, and other essential processes (Wang et al. 2020). This adaptability may contribute to the large population size of the Ruddy Shelduck.

In terms of gut microbiota composition, significant differences were observed among the bird species. In the Black‐necked Grebe, Greylag Goose, and Ruddy Shelduck, the most abundant bacterial phylum was Firmicutes. However, in the Relict Gull, Pseudomonadota was the dominant phylum. Studies have indicated that Firmicutes are more abundant in herbivorous animals, as they play a key role in breaking down cellulose into volatile fatty acids that can be utilized by the host, improving nutritional efficiency, modulating T‐cell responses, enhancing immunity, preventing intestinal inflammation, and maintaining gut microbial balance (Ley et al. 2006; Turnbaugh et al. 2009). Studies have shown that Relict Gulls mainly consume animal‐based food during the breeding season (Du et al. 2023), which may be the reason for the lower abundance of Firmicutes in their intestines compared to other bird species.

In the gut microbiota of the Greylag Goose and Ruddy Shelduck, Firmicutes accounted for more than 60% of the total composition, indicating that these two species primarily consume plant‐based foods and possess strong metabolic capacities. Previous studies reported that the gut microbiota of the Greylag Goose was dominated by Firmicutes (31.6%), Pseudomonadota (11.5%), Tenericutes (6.3%), and Fusobacteria (1.0%) (Wang et al. 2019). In this study, the Greylag Goose's gut microbiota was dominated by Firmicutes (62.9%), Pseudomonadota (31.7%), and Actinobacteriota (2.8%), with higher abundances of Firmicutes and Pseudomonadota compared to previous findings. This discrepancy may result from geographic differences. Notably, no Tenericutes were detected in the Greylag Goose's gut microbiota in this study, while Actinobacteriota were present at relatively high levels. Previous research indicated that Tenericutes are abundant in the gut microbiota of individuals with high‐fat diets (Fabbiano et al. 2018). Therefore, the absence of Tenericutes in the Greylag Goose in this study likely reflects differences in diet in this particular region.

Previous studies have shown that the most abundant microbial phyla in the gut of Ruddy Shelduck are Firmicutes (40.0%), Bacteroidetes (15.7%), Pseudomonadota (3.9%), and Fusobacteria (3.4%) (Wang et al. 2019). In this study, the dominant bacterial phyla in the gut of Ruddy Shelduck were Firmicutes (60.5%), Fusobacteriota (24.7%), Bacteroidota (8.2%), and Pseudomonadota (3.0%), which are consistent with the previous findings, though with slight differences in abundance. These results suggest that the gut microbiota of Ruddy Shelduck is relatively stable and less influenced by geographical factors. Previous studies have shown that in humans, an elevated Firmicutes/Bacteroidetes (F/B) ratio is associated with obesity (Panda et al. 2009; Lee et al. 2008). In our study, the F/B ratio of Ruddy Shelduck was 7.35, which was significantly higher than that reported in previous studies (2.57), indicating that the Ruddy Shelduck inhabiting the Ordos Relict Gulls National Nature Reserve may have a more efficient capacity to utilize food resources and convert them into energy.

In the gut of Black‐necked Grebe, Pseudomonadota had the lowest abundance, while the F/B ratio was the highest, suggesting that Black‐necked Grebe is well adapted to the region and can efficiently utilize food resources, indirectly reflecting a healthier physical state. In the gut of Greylag Goose, *Ligilactobacillus* accounted for 29.4%, which is a probiotic genus known to enhance the growth performance and antioxidative capacity of the gut, helping the host resist adverse environmental conditions (Yang et al. 2023). This indicated that the Greylag Geese in the Ordos Relict Gulls National Nature Reserve have good intestinal health.

Previous studies reported that the most abundant bacterial genus in the gut of Ruddy Shelduck was *Enterococcus* (16.7%) (Wang et al. 2019), which aligns with our results, where *Enterococcus* was also the dominant genus in the gut microbiota of Ruddy Shelduck, accounting for 22.0%. This further suggests that the gut microbiota of Ruddy Shelduck is relatively stable and less affected by external environmental factors. In the gut of Relict Gull, the most abundant bacterial genus was *Escherichia–Shigella*, accounting for 33.6%. The *Escherichia–Shigella* genus can promote inflammatory states in the body of mammals (Soares et al. 2012), and chronic infections with adhesive and invasive *Escherichia* species may lead to persistent peripheral inflammation (Ryu et al. 2012). But its role in birds remains unknown. In this study, it was found that the abundance in the gut of Relict Gull was relatively high, which indicated that the Relict Gull carries human pathogenic bacteria in their intestines. Therefore, relevant authorities should monitor these birds and implement protective measures to prevent the spread of pathogenic microorganisms. In the gut of Black‐necked Grebe, the most abundant bacterial genus was *Halomonas*, accounting for 22.5%. *Halomonas* is a common microorganism in high‐salinity environments (Chen et al. 2020), which may be associated with the nesting behavior and aquatic lifestyle of Black‐necked Grebe, as the water in this region has a high salt content. This also indirectly indicated that the gut microbiota of Black‐necked Grebe is more strongly influenced by its living environment.

Microorganisms residing in the gut can form symbiotic, synergistic, or antagonistic relationships to maintain the stability of the gut environment (Hu et al. 2016). In this study, the gut bacterial communities showed significant correlations, indicating that bacteria can influence each other to regulate the host's metabolism and physiological state. Correlation analysis revealed that *Marinobacterium* and *Halolactibacillus* had a highly significant positive correlation. Studies have shown that *Halolactibacillus* and *Marinobacterium* are halophilic and salt‐tolerant microorganisms commonly found in high‐salinity environments (Ryu et al. 2012). Both are capable of metabolizing aromatic compounds (Wang et al. 2020), indicating a symbiotic relationship, which is consistent with our results. *Acetoanaerobium* and *Proteiniclasticum* also exhibited a significant positive correlation. *Acetoanaerobium* is an anaerobic genus in the phylum Firmicutes that can degrade protein‐rich components (Böer et al. 2024), while *Proteiniclasticum* is also a protein‐degrading bacterium. These two bacteria with similar functions exhibited a symbiotic relationship.

KEGG predictions of gut microbial function showed that the gut microbiota was mainly associated with metabolism. At Level 1, the gut microbiota primarily participated in metabolic processes. At Level 2, they were mainly related to Global and overview maps, Carbohydrate metabolism, and Amino acid metabolism. At Level 3, Metabolic pathways were the main function. Further analysis of the gene content related to Human Diseases in the gut microbiota of these birds showed that it was higher in the gut microbiota of Relict Gull than in Ruddy Shelduck and Black‐necked Grebe, and it was higher in the gut microbiota of Greylag Goose than in Ruddy Shelduck. These findings indicate that the gut microbiota of birds in this area contains some human pathogens, warranting attention and preventive measures.

## 5 Conclusions

In summary, this study analyzed the gut microbial diversity and composition of four bird species inhabiting the Ordos Relict Gulls National Nature Reserve in Inner Mongolia, China. The results showed that the gut microbiota of Ruddy Shelduck exhibited the highest α‐diversity, while Greylag Goose had the lowest. The composition of gut microbiota varied significantly among the different bird species. The most abundant bacterial phylum in the guts of Black‐necked Grebe, Greylag Goose, and Ruddy Shelduck was Firmicutes, whereas the most abundant phylum in Relict Gull was Pseudomonadota. At the genus level, the dominant bacteria were *Halomonas* in Black‐necked Grebe, *Escherichia‐Shigella* in Relict Gull, *Ligilactobacillus* in Greylag Goose, and *Enterococcus* in Ruddy Shelduck. Correlation analysis revealed significant relationships among gut bacterial communities, indicating that bacteria can influence one another to regulate host metabolism and physiological states. This study offers important insights into the conservation of these bird species, particularly the Class I protected species, Relict Gull, and provides a foundation for the management of wild birds in this nature reserve.

## Author Contributions

**Mingxin Zou:** data curation (equal), resources (equal). **Xuanyu Li:** conceptualization (equal), formal analysis (equal), visualization (equal). **Chunyu Li:** formal analysis (equal), software (equal). **Hongda Pei:** investigation (equal), visualization (equal). **Ruobing Kang:** methodology (equal), supervision (equal). **Li Liu:** investigation (equal), writing – original draft (equal). **Li Gao:** conceptualization (equal), formal analysis (equal), software (equal), writing – original draft (equal), writing – review and editing (equal).

## Ethics Statement

The animal study was performed in accordance with the suggestions on animal care and ethics in China. Noninvasive techniques were used to collect fecal samples. The Animal Ethics and Welfare Committee of Baotou Teachers College approved the implementation of the project (No. AEWC‐BTTC2021‐002), and the management authorities of Ordos City in Inner Mongolia approved the sampling of fecal samples.

## Conflicts of Interest

The authors declare no conflicts of interest.

## Supporting Information

The following Supporting Information can be downloaded at: http://www.ncbi.nlm.nih.gov/bioproject/PRJNA1190508, http://www.ncbi.nlm.nih.gov/bioproject/PRJNA788023, and http://www.ncbi.nlm.nih.gov/bioproject/PRJNA956598.

## Supporting information


**Table S1.** Bacterial abundance at the phylum level in each group.

**Table S2.** Bacterial abundance at the genus level in each group.

## Data Availability

The datasets presented in this study are available at NCBI:PRJNA1190508 (SAMN45047391‐SAMN45047400), PRJNA788023 (SRX13401783, SRX13401784, SRX13401785, SRX13401786, SRX13401791, SRX13401792), PRJNA956598 (SRR24263419‐SRR24263422).
