# Supplementary material for: Comparative Analysis of Gut Bacteria of Four Waterbirds Species in Taolimiao‐Alashan Nur (T‐A Nur) in Erdos Relic Gull National Nature Reserve, Inner Mongolia, China
Source: Ecol Evol. 2025 May 13;15(5):e71432. doi: 10.1002/ece3.71432 (PMC12074897; doi:10.1002/ece3.71432)
Supplement: Supplementary file 1 — Table S1. Bacterial abundance at the phylum level in each group. [file ECE3-15-e71432-s002.docx]

Table S1. Bacterial abundance at the phylum level in each group.

| **phylum** | **Relative abundance(%)** | | | |
| --- | --- | --- | --- | --- |
|  | **PT** | **YO** | **CMY** | **HY** |
| Firmicutes | 44.20±11.38 | 40.29±13.60 | 60.49±30.99 | 62.85±15.89 |
| Pseudomonadota | 0.02±0.01^a^ | 46.06±18.43^b^ | 3.03±3.35^a^ | 31.69±15.84^b^ |
| Fusobacteriota | 3.19±4.27^a^ | 8.18±10.73^a^ | 24.67±22.47^b^ | 0.04±0.03^a^ |
| Bacteroidota | 0.15±0.05 | 2.83±3.50 | 8.23±11.4 | 0.33±0.22 |
| Actinobacteriota | 1.67±2.81^a^ | 1.48±1.78^ab^ | 1.04±1.18^ab^ | 2.80±1.50^b^ |
| Campylobacterota | 1.03±1.55 | 0.24±0.48 | 0.98±0.51 | 0.71±1.31 |
| Desulfobacterota | 0.10±0.05 | 0.07±0.09 | 0.64±1.14 | 0.04±0.03 |
| unclassified_Bacteria | 0.77±1.10^a^ | 0.11±0.15^a^ | 0.05±0.02^a^ | 1.14±0.67^b^ |
| Cyanobacteria | 0.19±0.21^a^ | 0.05±0.06^b^ | 0.02±0.03^b^ | 0.09±0.10^ab^ |
| Verrucomicrobiota | 0.50±0.67 | 0.13±0.15 | 0.29±0.59 | 0.03±0.02 |

Notes: YO:Relict Gulls; PT:Black-necked Grebe; HY:Greylag Goose; CMY:Ruddy Shelduck.
