# Supplementary material for: Comparative Analysis of Gut Bacteria of Four Waterbirds Species in Taolimiao‐Alashan Nur (T‐A Nur) in Erdos Relic Gull National Nature Reserve, Inner Mongolia, China
Source: Ecol Evol. 2025 May 13;15(5):e71432. doi: 10.1002/ece3.71432 (PMC12074897; doi:10.1002/ece3.71432)
Supplement: Supplementary file 2 — Table S2. Bacterial abundance at the genus level in each group. [file ECE3-15-e71432-s001.docx]

Table S2. Bacterial abundance at the genus level in each group.

| **genus** | **Relative abundance(%)** | | | |
| --- | --- | --- | --- | --- |
|  | **PT** | **YO** | **CMY** | **HY** |
| *Escherichia_Shigella* | 0.12±0.03^a^ | 33.60±24.17^b^ | 1.44±2.47^a^ | 0.51±0.28^a^ |
| *Ligilactobacillus* | 0.09±0.03^a^ | 4.42±6.15^a^ | 0.57±0.68^a^ | 29.41±13.94^b^ |
| *Paucibacter* | 0.03±0.04^a^ | 0.26±0.30^a^ | 0.09±0.08^a^ | 28.75±15.56^b^ |
| *Enterococcus* | 0.01±0.01^a^ | 0.17±0.23^a^ | 22.02±18.66^b^ | 1.32±2.37^a^ |
| *Halomonas* | 22.52±8.73^a^ | 0.03±0.02^b^ | 0.02±0.02^b^ | 0.05±0.05^b^ |
| *Catellicoccus* | 0.028±0.01^a^ | 17.70±13.84^b^ | 0.52±0.84^a^ | 0.00±0.00^a^ |
| *Fusobacterium* | 0.01±0.00^a^ | 0.66±0.97^a^ | 18.64±24.69^b^ | 0.04±0.02^a^ |
| *Turicibacter* | 0.00±0.00^a^ | 0.00±0.01^a^ | 0.10±0.09^a^ | 17.46±22.82^b^ |
| *Streptococcus* | 0.02±0.01^a^ | 0.49±0.67^a^ | 16.48±17.81^b^ | 0.31±0.35^a^ |
| *Cetobacterium* | 0.01±0.01 | 7.51±10.95 | 6.00±13.11 | 0.00±0.00 |

Notes: YO:Relict Gulls; PT:Black-necked Grebe; HY:Greylag Goose; CMY:Ruddy Shelduck.
